# Long pentraxin 3 as a marker of COVID-19 severity: evidences and perspectives

**DOI:** 10.11613/BM.2022.020901

**Published:** 2022-04-15

**Authors:** Roberto Assandri, Silvia Accordino, Ciro Canetta, Elisabetta Buscarini, Alessandro Scartabellati, Chiara Tolassi, Federico Serana

**Affiliations:** 1 Clinical Investigation Laboratory, Ospedale Maggiore di Crema, Crema, Italy; 2 High Care Internal Medicine Unit, fondazione IRCCS Ca’Granda Ospedale Maggiore Policlinico Milano, Milano, Italy; 3 Gastroenterology Unit, Ospedale Maggiore di Crema, Crema, Italy; 4 Pneumology 1 Unit, Ospedale Maggiore di Crema, Crema, Italy; 5 Clinical Chemistry Laboratory, Spedali Civili of Brescia, Brescia, Italy

**Keywords:** COVID-19, inflammation, biomarkers, intensive care units

## Abstract

**Introduction:**

Several laboratory tests are characteristically altered in Coronavirus Disease 2019 (COVID-19), but are not totally accurate in predicting the disease outcome. The long pentraxin 3 (PTX3) is quickly released directly at inflammation sites by many immune cell types. Previous studies have shown that PTX3 correlated with disease severity in various inflammatory conditions. Our study investigated the use of PTX3 as a potential marker of COVID-19 severity and compared its performance in detecting a more severe form of the disease with that of routine laboratory parameters.

**Materials and methods:**

Stored serum samples of RT-PCR confirmed COVID-19 cases that had been obtained at hospital admission were retrospectively analysed. Intensive care unit (ICU) stay was considered a surrogate endpoint of severe COVID-19. Pentraxin 3 was measured by a commercial enzyme-linked immunosorbent assay.

**Results:**

A total of 96 patients were recruited from May 1st, 2020 to June 30th, 2020; 75/96 were transferred to ICU. Pentraxin 3 was higher in ICU *vs* non-ICU patients (35.86 *vs* 10.61 ng/mL, P < 0.001). Univariate and multivariate logistic regression models demonstrated that the only significant laboratory predictor of ICU stay was PTX3 (OR: 1.68 (1.19-2.29), P = 0.003), after controlling for comorbidities. The Receiver Operator Characteristic curve analysis showed that PTX3 had a higher accuracy compared to C-reactive protein (CRP), lactate dehydrogenase (LD), ferritin in identifying ICU patients (AUC of PTX3 = 0.98; CRP = 0.66; LD = 0.70; ferritin = 0.67, P < 0.001). A cut-off of PTX3 > 18 ng/mL yielded a sensitivity of 96% and a specificity of 100% in identifying patients requiring ICU.

**Conclusion:**

High values of PTX3 predict a more severe COVID-19.

## Introduction

Many studies have shown that several routine laboratory tests are characteristically altered in Coronavirus Disease 2019 (COVID-19) (*1*). In a recent work we found that the use of Charlson comorbidity index and high C-reactive protein (CRP) at admission, identified patients with worse outcome (*2*). However, the use of comorbidity indexes was not totally appropriate to quickly identify patients with increased risk of evolution to a severe disease in the emergency room. C-reactive protein may be studied as a convenient alternative, but its diagnostic accuracy is variable and often unsatisfactory even in identifying COVID-19 cases during triage (*3*). Therefore, different inflammatory molecules are worth exploring as potential biomarkers of severity. Among them is the long pentraxin 3 (PTX3), which is a multimeric glycoprotein belonging to the pentraxin protein superfamily and is a key component of humoral innate immunity released in response to interleukin (IL)-1, tumor necrosis factor, and microbial agents (*4*). PTX3 can be rapidly released by both tissue cells and circulating leucocytes, whereas the short pentraxin prototype CRP needs to be synthesized by the liver in response to interleukin IL-6 during the acute-phase response (*4*). Therefore, PTX3 can be considered an earlier and “close-to-the-action” inflammation marker. Long pentraxin 3, indeed, is involved in resistance to selected pathogens and in the regulation of inflammation, including complement activation (*4*). Even though it is not currently measured in the routine setting, an increased PTX3 concentration has already been described as a promising biomarker of disease severity during several infection types and inflammatory conditions, including COVID-19 (*5*-*12*). Furthermore, a recently published study has also shown that PTX3 binds directly to the nucleocapsid portion of severe acute respiratory syndrome coronavirus 2 (SARS-CoV-2), thus confirming that it may play an active role in the pathogenesis of COVID-19 (*13*). For all these reasons, and keeping in mind that overinflammation is among the main features of the most severe COVID-19 cases, PTX3 quantification can be an ideal candidate biomarker to identify patients at higher risk of a severe disease (*14*). Therefore, our study evaluated the use of PTX3 blood concentration as a potential marker of COVID-19 severity and compared its performance with those of CRP and of other routinely determined laboratory inflammation markers.

## Materials and methods

### Subjects

A retrospective cross-sectional study was made by analysing all available stored samples from consecutive COVID-19 patients referring to Ospedale Maggiore di Crema from May 1^st^, 2020 to June 30^th^, 2020. Inclusion criteria were age ≥ 18 years and a confirmed SARS-CoV-2 infection by RT-PCR molecular testing. Samples were obtained within the first 24 hours following hospital admission. Intensive care unit (ICU) stay was considered a surrogate endpoint of disease severity. Stored serum samples from pre-COVID-19 outbreak healthy controls were also analysed. All analysis were performed using the residual material from the blood collected for routine tests. The study was approved by the hospital ethical review board.

### Methods

A serum separator tube was used for blood collection. Samples were allowed to clot for 30 minutes before centrifugation for 15 minutes at 1000xg. Sera were then collected, aliquoted and immediately stored at - 20 °C. Long pentraxin 3 serum concentrations were measured by a commercial sandwich enzyme-linked immunosorbent assay (ELISA) kit (Aiviscera Bioscience, Santa Clara, USA), based on recombinant human monoclonal antibodies. Sensitivity of the assay is 0.023 ng/mL. Samples were processed according to the manufacturer instructions and were diluted as needed when their concentration fell outside the standard range of the test. Routine laboratory markers were measured on Abbott Alinity Systems (Abbott, Chicago, USA) or Sysmex XN-20 analysers (Sysmex Corporation, Kobe, Japan).

### Statistical analysis

Normality of the distributions of continuous variables was tested by the Shapiro-Wilk test. Variables representing routine laboratory tests (neutrophils, lymphocytes, CRP, ferritin, lactate-dehydrogenase (LD)) did not follow a normal distribution, therefore their results were summarized as medians and interquartile ranges (IQR) and analysed using non-parametrical methods (Mann-Whitney test for median comparison, Spearman test for correlations). Long pentraxin 3 concentrations and age followed a Gaussian distribution therefore data were analysed by t-test in the case of mean comparison between two groups (male *vs* female). The Kruskal-Wallis non-parametrical test (followed by the Dunn’s *post-hoc* test) was used for comparisons between the three groups (controls *vs* ICU *vs* non-ICU), because the Bartlett’s test failed to show a homogeneous variance. Correlations between age and PTX3 were analysed by Pearson’s coefficient. Proportions were compared by the Fisher’s exact test. Logistic regression models were fit to assess baseline factors predicting ICU stay, as a surrogate endpoint of disease severity. Variables whose logit-coefficients showed a P-value < 0.10 in univariate models were then fit into multivariate models. Receiver operating characteristic (ROC) curve analysis was performed to quantify the accuracy of PTX3 *vs* routine laboratory markers in identifying ICU patients. Analysis were performed using Stata v. 16.0 (StataCorp LLC, College Station, USA). Results were considered significant when P < 0.05, unless differently specified.

## Results

Ninety six patients matched the inclusion criteria; 75/96 were transferred to ICU. Patients who died were 14/96 (Table 1). Patients’ demographic characteristics and routine laboratory test results are reported in Table 1, showing that, among them, only CRP, ferritin and LD concentrations resulted significantly higher in patients transferred to the ICU compared to non-ICU patients.

**Table 1:** Demographic features and routine laboratory test results of enrolled patients

	**Non-ICU** **(N = 21)**	**ICU** **(N = 75)**	**Total** **(N = 96)**	**P**
Age, years	66 (48-88)	63 (36-83)	64 (36-88)	0.265
Gender, M (N, proportion)	16 (0.76)	57 (0.76)	73 (0.76)	0.617
Comorbidities, Yes (N, proportion)	8 (0.38)	53 (0.71)	61 (0.64)	0.010
Deceased, Yes (N, proportion)	1 (0.05)	13 (0.17)	14 (0.15)	0.096
Neutrophils, x 10^9^/L	6.7 (5.3)	8.2 (5.3)	8.1 (5.4)	0.291
Lymphocytes, x 10^9^/L	0.8 (0.5)	0.7 (0.3)	0.7 (0.4)	0.309
CRP, mg/L	35.1 (51.9)	69.9 (132.4)	53.9 (110.2)	0.022
Ferritin, μg/L	535 (619)	1014 (1500)	937 (1389)	0.015
LD, U/L	298 (89)	380 (164)	365 (175)	0.004
IL-6, pg/mL	20.01 (33.90)	12.40 (20.50)	12.65 (26.65)	0.551
Age is presented as median (range). Continuous variables are described in terms of median (IQR), categorical variables are reported as numbers (proportions). ICU - Intensive care unit. Comorbidities include malignancies, diabetes or hypertension. CRP – C-reactive protein. LD – lactate dehydrogenase. IL-6 – interleukin-6.				

**Figure 1:**
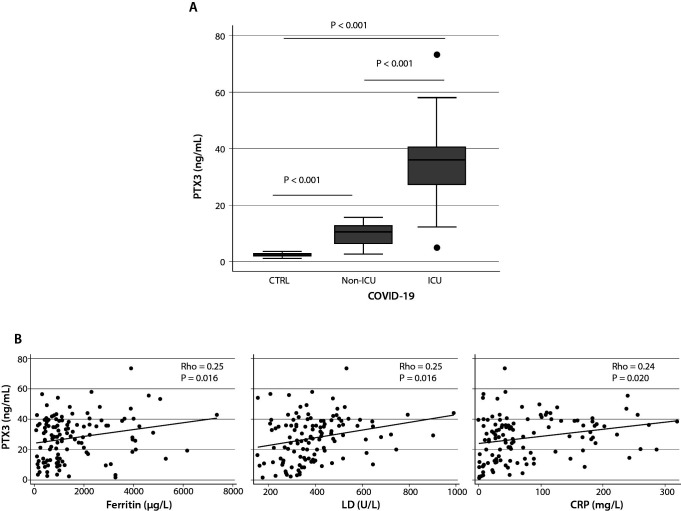
PTX3 concentration and correlations with routinely-used inflammatory markers in COVID-19 patients. (A) Box and whiskers plot showing PTX3 concentrations in controls, ICU and non-ICU patients. (B) PTX3 correlations with the other routinely-used inflammatory markers (lines obtained by linear regression). ICU – Intensive care unit. CTRL – controls. PTX3 – long pentraxin 3. LD – lactate dehydrogenase. CRP – C-reactive protein.

To evaluate the role of PTX3, we first verified that PTX3 did not change with gender in none of the groups (Supplementary material), because there was an imbalance in the subjects’ gender in patients *vs* controls (females 23/96 *vs* 26/50 respectively; P = 0.001). Furthermore, we observed that PTX3 had a non-significant, slightly decreasing trend with age (r = - 0.20, P = 0.055) in patients, but not in controls (Supplementary material). However, even though patients were significantly older than controls (64 *vs* 39 years, P < 0.001), PTX3 in COVID-19 patients was higher in respect to controls (median value 31.32 *vs* 2.30 ng/mL, P < 0.001) (Figure 1A). More importantly, although PTX3 was only weakly correlated with the other routinely-used inflammatory markers (ferritin, LD, CRP, Figure 1B), higher PTX3 concentrations appeared tightly linked to the need of ICU. Indeed, patients admitted to ICU showed higher PTX3 concentrations compared to non-ICU patients (median value 35.86 *vs* 10.61 ng/mL, P < 0.001) (Figure 1A).

**Figure 2:**
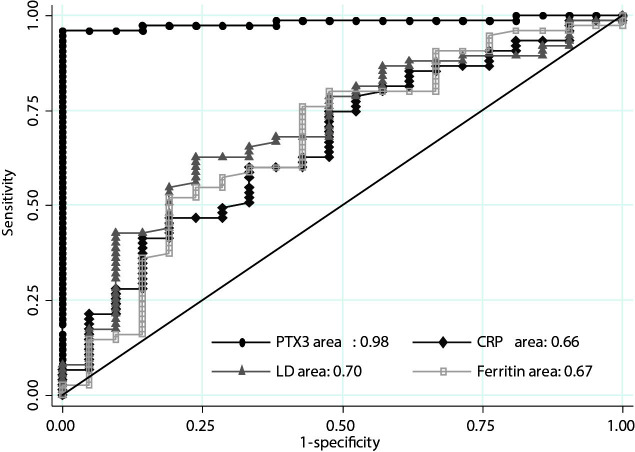
ROC Curve analysis of PTX3 compared to routinely-used inflammatory markers. All areas under the curves were significantly different from 0.50. PTX3 was compared to laboratory markers that were increased in ICU *vs* non-ICU patients. ICU - Intensive care unit. CRP – C-reactive protein. LD – lactate dehydrogenase. PTX3 - long pentraxin 3.

To confirm the usefulness of PTX3 in predicting ICU stay, univariate and multivariate logistic regression models were fitted (Table 2). Results showed that, after controlling for comorbidities, PTX3 was the only significant predictor among inflammation markers (odds ratio (OR) with 95% confidence interval (95%CI): 1.68 (1.19-2.29, P = 0.003). According to the multivariate model, each 1 ng/mL increase in PTX3 concentration predicted a 65% higher risk of ICU stay. ROC curve analysis showed that PTX3 had a significantly better performance in identifying ICU *vs* non-ICU patients compared to the other routine inflammatory markers (Figure 2) (Area Under the Curve (AUC) of PTX3 = 0.98 (0.96-1.00) *vs* CRP = 0.66 (0.53-0.79) *vs* LD = 0.70 (0.58-0.83) *vs* ferritin = 0.67 (0.54-0.81), P < 0.001). A cut-off of PTX3 > 18 ng/mL yielded a sensitivity of 96% and a specificity of 100% in identifying patients requiring ICU, with a 97% of patients correctly classified. Furthermore, we noted that 12/13 dead patients had PTX3 serum concentrations higher than 18 ng/mL.

**Table 2:** Logistic regression modelling of the risk of being in ICU

	**Univariate (N = 96)**				**Multivariate (N = 96)**			
**Predictors**	**Coefficient**	**OR**	**95%CI**	**P**	**Coefficient**	**OR**	**95%CI**	**P**
Age, years	- 0.029 (0.024)	0.97(0.02)	0.93-1.01	0.231	- 0.057 (0.062)	0.94 (0.06)	0.83-1.06	0.360
PTX3, ng/mL	0.421 (0.123)	1.52 (0.19)	1.20-1.94	0.001	0.501 (0.168)	1.65 (0.28)	1.19-2.29	0.003
Comorbidities	1.365 (0.516)	3.92 (2.02)	1.42-10.7	0.008	3.809 (1.841)	45.12 (83.07)	1.22-1665	0.039
Ferritin, μg/L	0.000 (0.000)	1.00 (0.00)	0.99-1.00	0.121	-	-	-	-
LD, U/L	0.006 (0.003)	1.01 (0.00)	1.00-1.01	0.018	-	-	-	-
CRP, mg/L	0.008 (0.004)	1.01 (0.00)	0.99-1.01	0.051	-	-	-	-
Pseudo R^2^	-							
Standard errors are presented in parenthesis. OR – odds ratio = e^(Coefficient)^. 95%CI – 95% confidence interval. PTX3 - long pentraxin 3. CRP – C-reactive protein. LD – lactate dehydrogenase.								

## Discussion

We previously underlined the concept that few parameters (neutrophils, lymphocytes, CRP, LD) could be used to assess the presence and even the severity of COVID-19 during triage in the emergency room (*15*). However, these biomarkers are not completely accurate (*3*). The present study was performed to investigate the expression of PTX3 during COVID-19 infection and its predictive potential in respect to the clinical outcome of developing a severe disease (defined as need of ICU or ICU stay). The strongest evidence in our work is represented by the very high PTX3 serum concentration in ICU-transferred patients compared to non-ICU (median 35.86 *vs* 10.61 ng/mL), which suggests that PTX3 can be considered a marker of COVID-19 severity. Multivariate analysis, which was performed to control for comorbidities and for possible confounders, corroborated this evidence of PTX3 as an independent predictor of severity (OR: 1.68 (1.19-2.29), P = 0.003), indicating that each 1 ng/mL increase in PTX3 concentration predicts a 65% higher risk of ICU need. Moreover, in our cohort, PTX3 accuracy in identifying ICU patients (AUC = 0.98 (0.96-1.00)) was much higher than that of CRP, ferritin and LD and the cut-off of PTX3 > 18 ng/mL yielded a 96% sensitivity and 100% specificity in identifying patients requiring ICU. Despite the main limitations of our study, which are the small sample size, the absence of a validation cohort for logistic regression, and its retrospective, cross-sectional design, our results seem perfectly in line with the few already published about PTX3 as marker of worse outcome or severity in COVID-19 (5-12). For example, one of these studies showed that PTX3 predicts 28-day mortality in COVID-19 better than conventional markers and that PTX3 was higher in ICU patients *vs.* medical wards, with concentrations that were strikingly similar to those reported in the present study (21.0 ng/mL in ICU *vs* 12.4 ng/mL in non-ICU), despite the lack of any assay harmonization (*6*). Another study reported higher PTX3 in ICU *vs* non-ICU patients, and two more studies a better performance of PTX3 in respect to CRP in predicting mortality (*9*, *12*, *13*). The strength of PTX3 association with COVID-19 mortality is underlined by the results of a study that followed a totally different methodological approach: a large proteomic study employing machine learning found that PTX3 was as one of the proteins most strongly associated with mortality among 1472 unique proteins measured, outperforming most measured cytokines and chemokines (*11*). Finally, the role of PTX3 and of the humoral innate immunity during SARS-CoV-2 infection is further supported by a recent investigation showing that PTX3 directly binds SARS-CoV-2 nucleocapsid (*13*). This new evidence, suggests the hypothesis of complement cascade involvement in the overinflammation usually observed in severe COVID-19 (*4*, *14*). Indeed, it is well-known that inflammation and complement activation are a double-edged sword. Therefore, the high concentrations of PTX3 measured in severe COVID-19 cases might reflect an underlying misregulated, uncontrolled inflammation (*4*, *14*).

In conclusion, our data contribute to the accumulating evidence that PTX3 blood concentration may serve as an early marker of COVID-19 severity and support the need of larger, prospective studies that would hopefully pave the way to the use of PTX3 in the routine clinical practice.
